# Neutralizing and binding antibody responses to SARS-CoV-2 with hybrid immunity in pregnancy

**DOI:** 10.1038/s41541-024-00948-3

**Published:** 2024-08-27

**Authors:** Lin Li, Yusuke Matsui, Mary K. Prahl, Arianna G. Cassidy, Yarden Golan, Unurzul Jigmeddagva, Nida Ozarslan, Christine Y. Lin, Sirirak Buarpung, Veronica J. Gonzalez, Megan A. Chidboy, Emilia Basilio, Kara L. Lynch, Dongli Song, Priya Jegatheesan, Daljeet S. Rai, Balaji Govindaswami, Jordan Needens, Monica Rincon, Leslie Myatt, Taha Y. Taha, Mauricio Montano, Melanie Ott, Warner C. Greene, Stephanie L. Gaw

**Affiliations:** 1 Division of Maternal-Fetal Medicine, Department of Obstetrics, Gynecology, and Reproductive Sciences, University of California San Francisco, San Francisco, CA USA; 2 Department of Obstetrics, Gynecology, and Reproductive Sciences, Center for Reproductive Sciences, University of California San Francisco, San Francisco, CA USA; 3 Gladstone Institute of Virology, San Francisco, CA USA; 4 Michael Hulton Center for HIV Cure Research at Gladstone, San Francisco, CA USA; 5 Department of Pediatrics, University of California San Francisco, San Francisco, CA USA; 6 Division of Pediatric Infectious Diseases and Global Health, University of California San Francisco, San Francisco, CA USA; 7 Department of Bioengineering and Therapeutic Sciences, University of California San Francisco, San Francisco, CA USA; 8 Department of Laboratory Medicine, University of California San Francisco, San Francisco, CA USA; 9 Division of Neonatology, Department of Pediatrics, Santa Clara Valley Medical Center, San Jose, CA USA; 10 Stanford-O’Connor Family Medicine Residency Program, Division of Family Medicine, Stanford University, Palo Alto, CA USA; 11 Division of Neonatology, Department of Pediatrics, Marshall University Joan C Edwards School of Medicine, Huntington, WV USA; 12 Department of Obstetrics and Gynecology, Marshall University Joan C Edwards School of Medicine, Huntington, WV USA; 13 Department of Obstetrics and Gynecology, Oregon Health & Science University, Portland, OR USA; 14 Department of Medicine, University of California San Francisco, San Francisco, CA USA; 15 Departments of Microbiology and Immunology, University of California San Francisco, San Francisco, CA USA

**Keywords:** Antibodies, Viral infection, RNA vaccines, SARS-CoV-2

## Abstract

Hybrid immunity against SARS-CoV-2 has not been well studied in pregnancy. We conducted a comprehensive analysis of neutralizing antibodies (nAb) and binding antibodies in pregnant individuals who received mRNA vaccination, natural infection, or both. A third vaccine dose augmented nAb levels compared to the two-dose regimen or natural infection alone; this effect was more pronounced in hybrid immunity. There was reduced anti-Omicron nAb, but the maternal-fetal transfer efficiency remained comparable to that of other variants. Vaccine-induced nAbs were transferred more efficiently than infection-induced nAbs. Anti-spike receptor binding domain (RBD) IgG was associated with nAb against wild-type (Wuhan-Hu-1) following breakthrough infection. Both vaccination and infection-induced anti-RBD IgA, which was more durable than anti-nucleocapsid IgA. IgA response was attenuated in pregnancy compared to non-pregnant controls. These data provide additional evidence of augmentation of humoral immune responses in hybrid immunity in pregnancy.

## Introduction

The safety and effectiveness of vaccines during pregnancy have been demonstrated in numerous studies^1–3^. A robust humoral immune response is activated after both mRNA COVID-19 vaccination and SARS-CoV-2 infection. Passive immunity through *in utero* antibody transfer to the fetus plays an important role in protection against COVID-19 during the first 6 months of life^4–7^. In late 2021, the highly transmissible Omicron (B.1.1.529) variant supplanted the Delta (B1.617.2) variant to become the predominant strain in the United States within one month. Novel mutations throughout the genome of the Omicron variant and its subvariants such as BA.2, BA.4, and BA.5 confer significant immune escape properties from both vaccine- and infection-induced immunity, resulting in the highest levels of COVID-19 case rates to date^8^.

In pregnancy, the Omicron wave was associated with high rates of infection but lower rates of severe disease as compared to the Delta variant, which was particularly virulent in pregnant individuals^9^. Infants were also more vulnerable to the Omicron variant—young infants 0–6 months of age were hospitalized at a rate similar to adults 65 years and older^10^. An in-depth understanding of functional antibody responses in the maternal-fetal dyad with evolving population levels of immunity due to vaccination and prior infection is needed to optimize the protection of pregnant individuals and their young infants against evolving COVID-19 variants.

Neutralizing antibodies (nAbs) are the most effective and specific defense against SARS-CoV-2 infection through targeting the receptor binding domain (RBD) on Spike (S) glycoprotein and disrupting virus-receptor engagement and subsequent cell entry^11,12^. Over 30 mutations within the S-protein of the Omicron variant (and 15 mutations on the RBD) contribute to its resistance to neutralization by pre-existing antibodies^13^. Pregnancy may induce further reductions in neutralization capacity^14^. S-protein binding antibodies are frequently tested in studies of serologic response or seroprevalence; of these, just a small proportion specifically binds the RBD to neutralize the virus against cell entry^15,16^. Binding antibodies that target the nucleocapsid (N) protein help to distinguish between infection- and vaccine-induced immunity^17^. The positive correlation between binding and nAbs has been demonstrated in non-pregnant populations, and anti-S IgG binding assays are commonly used to predict protective immunity against COVID-19^18,19^. However, it remains unclear whether the correlation between binding and nAbs extends to pregnant individuals.

As the mRNA-1273 and BNT162b2 vaccines deliver mRNA encoding only the S-protein of SARS-CoV-2^20^, vaccination is expected to elicit antibodies against RBD, but not N-protein. Both vaccination and infection induce IgA antibodies, secreted in saliva and breast milk to confer mucosal immunity^21–25^. While plasma levels of anti-SARS-CoV-2 IgA have been reported in pregnant individuals, these studies have not investigated the specificity and durability of IgA responses in detail^26–29^. IgA antibodies are an important component of mucosal immunity and may be more efficiently induced in response to natural infection^30^. IgA cannot cross the placental barrier and is primarily passed to the infant through breast milk. The data for the characteristics and dynamics of IgA produced during pregnancy is needed to optimize future vaccination development for the maternal-infant dyad, particularly in the era of widespread hybrid immunity.

Natural immunity, vaccine-induced, and hybrid immunity to SARS-CoV-2 confer varying levels of protection against severe disease^31^. Comprehensive studies of these different types of immunity in pregnancy and lactation are limited. To address these questions, we examined the nAb activity against various strains, including Omicron in pregnant individuals infected or vaccinated with wild-type (WT) (Wuhan-Hu-1) SARS-CoV-2 sequences. We also quantified the transfer of these antibodies to the fetus. The dynamics of N- and RBD-specific IgG and IgA antibody levels after sequential exposures to SARS-CoV-2 antigen (via vaccination or infection) were also evaluated. Finally, we described the durability of anti-N and anti-RBD IgA after vaccination and infection.

## Results

### nAbs in pregnant individuals with varied immunization status

**Fig. 1:**
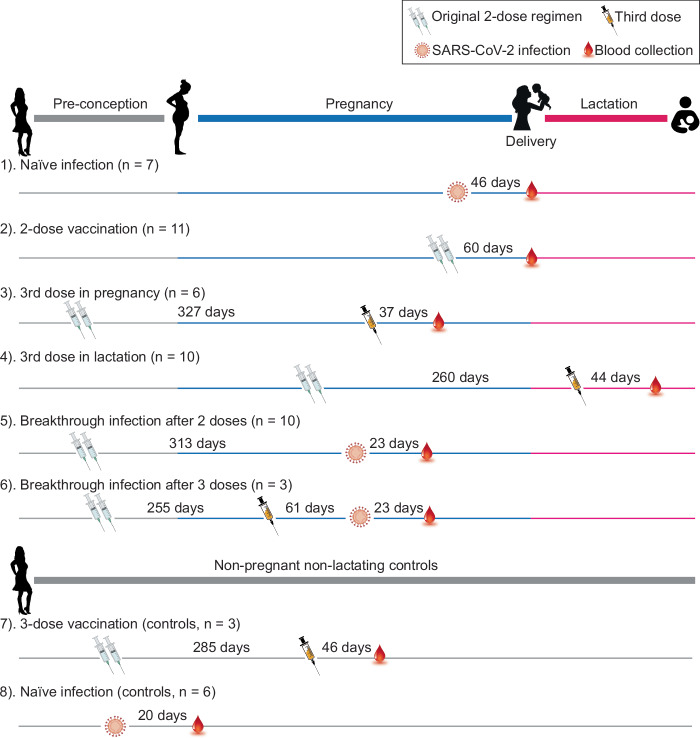
Timeline of vaccination, infection, and sampling in each group. Participants with different exposure types and numbers are divided into eight groups. Days indicate median time intervals between each antigen exposure and sample collection. Maternal blood samples were collected at the time of delivery in Group 1 and Group 2. All participants delivered at full term (≥37 weeks gestational age). Group 3 donated samples ~37 days after the third vaccine dose in pregnancy, prior to delivery. All participants in Group 4 were lactating at the time of the third-dose vaccination and sample collection. Groups 5 and 6 experienced breakthrough infections during pregnancy and samples were collected ~23 days after diagnosis. The two control groups (Groups 7 and 8) were non-pregnant, non-lactating females of reproductive age who were vaccinated or infected, respectively.

**Fig. 2:**
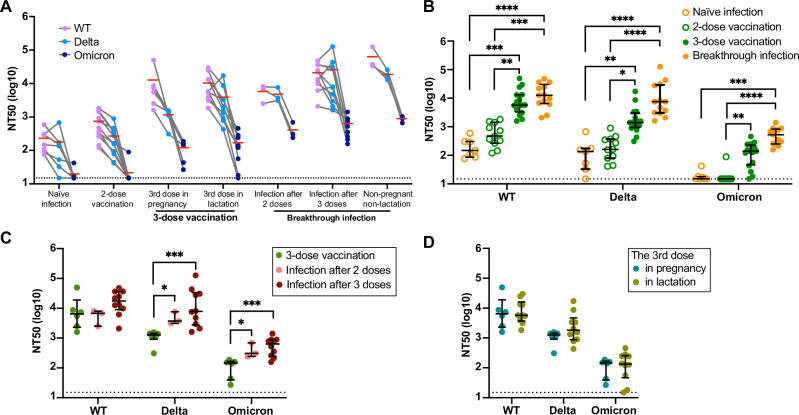
Neutralizing activity of maternal plasma from pregnant individuals with different immunization statuses. **A** Plasma neutralization titers were measured by a SARS-CoV-2 Spike pseudotyped virion assay for WT (Wuhan Hu-1), Delta, and Omicron BA.1 Spike protein sequences. Red horizontal lines for each group denote the mean titer. **B** NT50 values in individuals with naïve infection, 2-dose vaccination, 3-dose vaccination, and breakthrough infection (after two or three vaccine doses). **C** Comparison of the NT50 values elicited by 3-dose vaccination, breakthrough infections after 2-dose, and breakthrough infection after 3-dose vaccination in pregnant individuals. **D** Comparison of the NT50 values elicited by 3-dose vaccination with the third dose administered during pregnancy vs lactation. The horizontal dotted line represents the positive cutoff NT50 value of 15. Black bars represent median value and interquartile ranges (IQRs). *P < 0.05, **P < 0.01, ***P < 0.001, ****P < 0.0001 by Mann–Whitney test or Kruskal–Wallis test with Dunn’s multiple comparisons test. The black error bars depict the median ± IQR.

To evaluate the nAb responses after different types and timing of exposure, we compared NT50 values among pregnant and non-pregnant individuals with naïve infection, two doses of vaccine, three doses of vaccine, and breakthrough infection (after two or three doses) (Group 1–7 in Fig. 1). The neutralizing activity against the SARS-CoV-2 WT, Delta, and Omicron (BA.1) variants was evaluated in plasma samples from 50 individual participants. Neutralizing activity against the Omicron variant was dramatically reduced in all participants compared to the WT and Delta variants (Fig. 2A). The mean NT50 titer was reduced by 48-fold (11–106 fold, *P* < 0.001) compared to WT in all groups. Compared to the Delta variant, the mean NT50 against Omicron was 18-fold lower (9–41 fold, *P* < 0.001).

We then compared the NT50 titers against each variant by exposure history (Fig. 2B). There was no difference in nAb activity against all three variants between naïve infection and two vaccine doses. Immune boosting through the third mRNA vaccine dose significantly increased nAb titers against all three variants compared to the 2-dose regimen (15.3-fold, 10.9-fold, and 6.9-fold for each variant, respectively; *P* < 0.05). Breakthrough infection (during the Omicron wave) elicited the highest nAb levels against all three variants than naïve infection (76.5-fold, 116.8-fold, and 29.2-fold; *P* < 0.001). We found no difference in the nAb responses for any of the variants from breakthrough infection after two or three vaccine doses (Fig. 2C). When comparing the NT50 in individuals with ≥3 exposures to SARS-CoV-2 antigens, hybrid immunity after two vaccine doses induced higher NT50 against the Delta and Omicron variants than immunity after three doses of vaccine alone (4.2-fold and 3.5-fold, *P* < 0.05). Breakthrough infection after three doses also elicited higher levels of nAb than 3-dose vaccination only (22.1-fold and 5.2-fold, *P* < 0.001 (Fig. 2C).

Due to the timing of the third dose approval in the US during our study period, pregnant participants seldom received all three doses during their pregnancy. Participants typically initiated the first dose during their pregnancy and then received the third dose after delivery or completed the primary two doses prior to conception and received the third dose during pregnancy. We compared responses between those who received the third dose in pregnancy vs in lactation—there was no difference in nAb levels (Fig. 2D).

### Maternal-fetal transfer of maternal antibodies

**Fig. 3:**
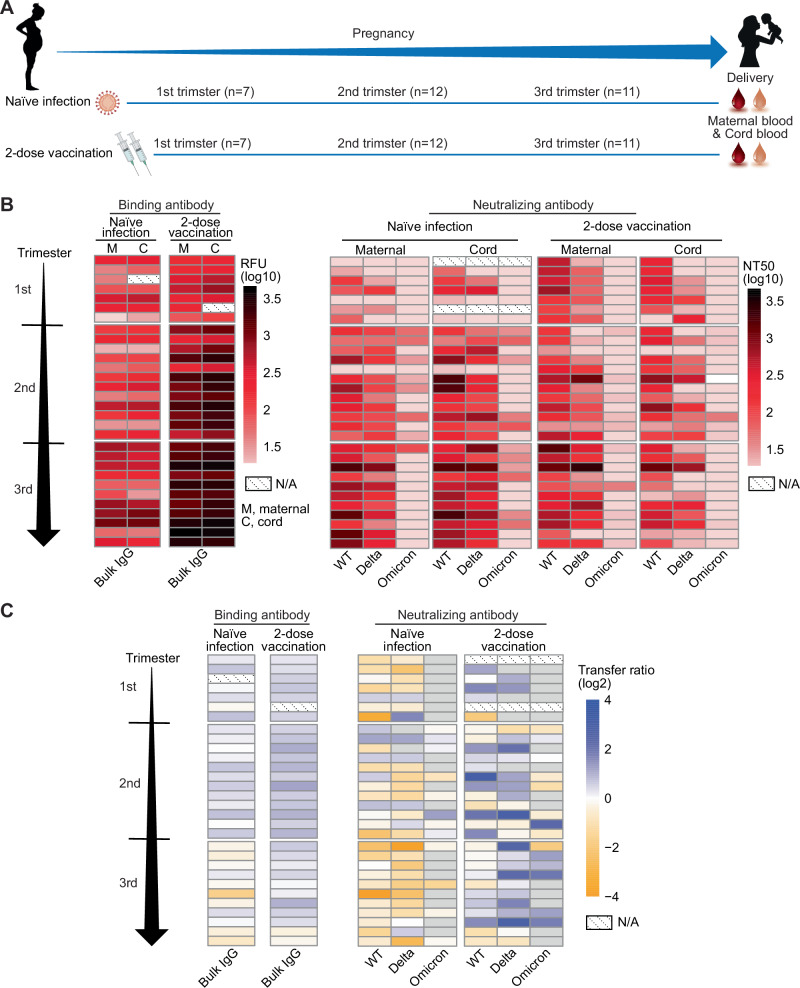
Maternal-fetal transfer of maternal neutralizing and binding antibodies in pregnant individuals with original 2-dose vaccine series and naïve infection. **A** Participant cohort. Pregnant participants with naïve infection (*n* = 30) or 2-dose vaccination (*n* = 30) were matched by gestational age of exposure. Paired maternal and cord blood samples were collected at the time of delivery. **B** Binding antibodies (left) and neutralizing activities against the WT, Delta, and Omicron variants (right) in paired maternal and cord blood at delivery. **C** Maternal-cord blood transfer ratio of binding antibodies (left) and neutralizing activities against the WT, Delta, and Omicron variants (right), shown in log2. The timing of exposure between infected and vaccinated individuals was matched within 1 week gestational age. All participants were grouped by trimester of the first exposure and ordered by increasing gestational week.

To explore the impact of gestational timing of exposure and the intrauterine transfer of maternal neutralizing and binding antibodies, we analyzed the nAbs against the three variants and bulk IgG in matched maternal and cord blood samples at the time of delivery in naïve infection and 2-dose vaccination. Thirty pregnant individuals who were infected during the WT and Alpha waves (March 2020 to January 2021) and 30 pregnant individuals who completed the primary 2-dose vaccine series before delivery were matched by gestational age of exposure (±1 week) and analyzed by trimester of exposure (Fig. 3A). Two-dose vaccination induced a much higher level of anti-N and RBD binding IgG in both maternal and cord blood than naïve infection, especially in the second and third trimesters of exposure (Fig. 3B). Naïve infection resulted in lower levels of nAbs against the WT and Delta variants in the cord than in the maternal blood (*P* < 0.05 and *P* < 0.01) (Supplementary Fig. 1A). In contrast, nAbs were comparable between maternal and cord plasma in the 2-dose vaccination group, with a higher level of the nAb against the Delta in cord blood than in maternal blood (*P* < 0.001). When analyzed by trimester of exposure, vaccination in the third trimester resulted in higher nAb against Delta in cord blood; no neutralizing activity against Omicron was detected in either maternal or cord blood after first-trimester vaccination and infection (Supplementary Fig. 1B). In cord blood, nAb against the WT and Delta variants gradually increased from mothers vaccinated in the first, second, and third trimesters. NAb against the Omicron variant was detected in 41.6% (5/12) of cord blood samples following second-trimester vaccination and 54.5% (6/11) following third-trimester vaccination; anti-Omicron nAbs were detected in 50% (6/12) and 18.1% (2/11) of cord blood after second and third-trimester infections, respectively (Supplementary Fig. 1C).

The transfer ratio was calculated to quantify the efficiency of antibody transfer (calculated by dividing the cord antibody level by the maternal antibody level) in dyad samples (Fig. 3C). Vaccination was associated with a higher transfer ratio of both the binding and nAbs, particularly in the nAb against WT (2.2-fold higher, *P* < 0.01) and Delta (3.1-fold higher, *P* < 0.0001) compared to naïve infection. Although nAb against the Omicron dropped dramatically in maternal and cord blood compared to the WT and Delta variants, the transfer ratio did not differ between variants (Supplementary Fig. 2A). Trimester of exposure did not impact variant-specific transfer ratios (Supplementary Fig. 2B). However, there appeared to be individual-level variability in the transfer efficiency of variant-specific nAbs after both vaccination and infection (Fig. 3C).

### N- and RBD-specific IgG antibodies and correlation with nAbs

**Fig. 4:**
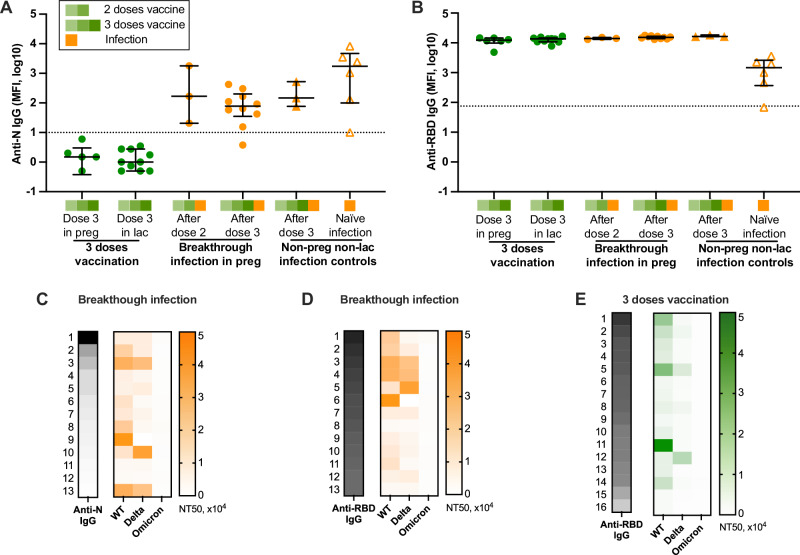
Anti-SARS-CoV-2 N and RBD IgG antibodies, and correlations between IgG (MFI) and neutralizing antibody titer (NT50). **A** Anti-N and **B** anti-RBD IgG antibodies in individuals with 3-dose vaccination or breakthrough infection. Non-pregnant and non-lactating women with infection were used as controls. The dotted line represents the positive cutoff value. Preg pregnancy, lac lactation. The black error bars depict the median ± IQR. **C**–**E** Ordered by decreasing anti-N or anti-RBD IgG levels (gray), NT50 against the WT, Delta, and Omicron variants in each individual following breakthrough infection (**C**, **D**, orange) or 3-dose vaccination (**E**, green) are indicated with the color scale. The left numbers represent the participant number (breakthrough infection *n* = 13; 3-dose vaccination *n* = 16).

SARS-CoV-2 specific binding antibodies are commonly used as a measure of immunity, due to the limited availability of neutralizing assays in most settings^32–38^. To gain a comprehensive understanding of the anti-SARS-CoV-2 humoral IgG response, we evaluated IgG antibodies against N and RBD after the third dose of the mRNA vaccine or breakthrough infection (Fig. 4). As expected, individuals without prior SARS-CoV-2 infection had no detectable anti-N IgG (Fig. 4A). In infected individuals, there was no difference in the anti-N IgG levels between breakthrough infection after two vs three doses; in one individual who was tested 12 days after infection, the level was equivocal (just below the positive cutoff). In non-pregnant and non-lactating controls (*n* = 6), results were similar; one participant tested 9 days after infection had equivocal results (Fig. 4A). RBD-specific IgG antibody levels after 3-dose vaccination (*n* = 16) were similar to those after breakthrough infection (*n* = 13). All individuals with 3-dose vaccination or breakthrough infection had much higher levels of anti-RBD IgG than non-pregnant controls with naïve infection (Fig. 4B).

We next explored associations between binding antibodies and nAbs to determine whether IgG antibodies can accurately predict neutralizing activity. Individuals with breakthrough infection or 3-dose vaccination were ordered by decreasing levels of anti-N or -RBD IgG and correlated with nAb results against WT, Delta, and Omicron strains (Fig. 4C–E). Neutralizing activities were more strongly correlated with anti-RBD than with anti-N IgG. Higher levels of anti-RBD IgG were associated with increased nAb against WT and Delta variants in both the breakthrough and vaccinated groups (Fig. 4D, E). Omicron-specific antibodies were low in all groups. Spearman’s rank correlation analyses between NT50 values of nAb and MFI levels of IgG antibodies for each variant revealed that only nAb against WT elicited by breakthrough infection is significantly correlated with RBD-specific IgG (*r* = 0.70, *P* = 0.01) (Supplementary Fig. 3).

### Anti-N and anti-RBD specific IgA antibodies in individuals with varied exposure history

**Fig. 5:**
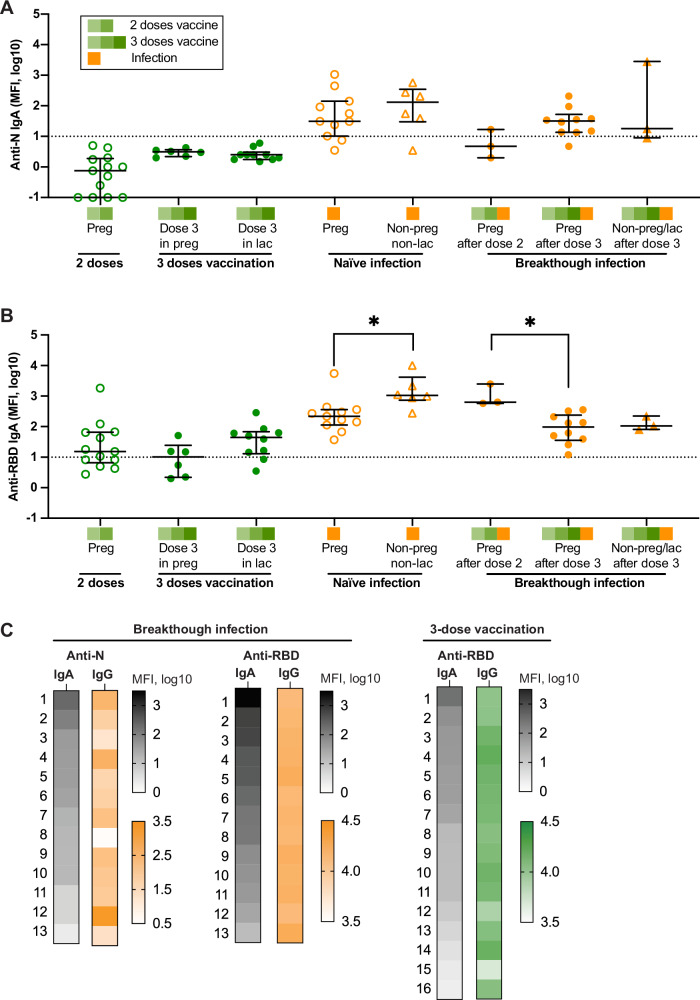
Anti-SARS-CoV-2 N and RBD IgA antibodies in plasma from individuals with varied exposure history. **A** Anti-N and **B** anti-RBD IgA antibodies in pregnant, lactating, and non-pregnant/non-lactating individuals with varied exposure history. The dotted line represents the positive cutoff value. Preg, pregnancy; lac, lactation. *P < 0.05 by Mann–Whitney test or Kruskal–Wallis test. The black error bars depict the median ± IQR. **C** Ordered by decreasing anti-N or anti-RBD IgA antibodies (gray), corresponding IgG antibodies for each individual are shown in color scale. The left numbers represent the participant number (breakthrough infection *n* = 13; 3-dose vaccination *n* = 16).

N-specific IgA was not detected in vaccinated individuals, while 78% (26/33) of infected individuals had detectable plasma anti-N IgA. Naïve infection and breakthrough infection elicited similar levels of anti-N IgA antibodies in pregnant and non-pregnant individuals (Fig. 5A). Anti-RBD IgA antibodies were detected in 65.5% (19/29) of vaccinated individuals, while it was present in 100% (33/33) of infected individuals regardless of exposure history (Fig. 5B). After naïve infection, non-pregnant, non-lactating individuals had higher anti-RBD IgA antibodies than pregnant individuals (3.8-fold, *P* = 0.01). In breakthrough infection groups, pregnant individuals infected after two doses had higher anti-RBD IgA than those after three doses (9.1-fold, *P* = 0.02), while there was no significant difference in breakthrough infection after three vaccine doses, in both pregnant and non-pregnant individuals. There was no correlation between levels of IgA and IgG in breakthrough infection or 3-dose vaccination (Fig. 5C). Spearman’s rank correlation analyses showed no significant correlation between anti-RBD or anti-N IgA and IgG in vaccination and infection groups (Supplementary Fig. 4A) or between IgA and nAb against each variant (Supplementary Fig. 4B).

**Fig. 6:**
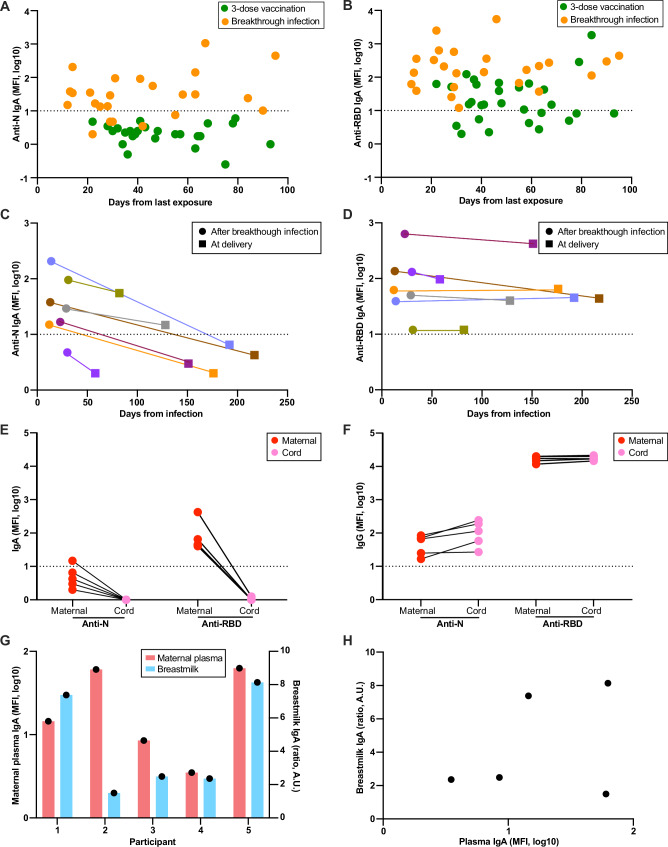
Kinetics of IgA production and decay. **A** Anti-N and **B** anti-RBD IgA antibody levels are shown by the timing from exposure (the third dose of vaccine or breakthrough infection). **C**, **D** Maternal blood samples were collected after 12-31 days after SARS-CoV-2 breakthrough infection and at delivery (*n* = 7). Anti-N (**C**) and anti-RBD (**D**) IgA antibody levels are plotted by the timing of PCR positivity to show the durability of IgA antibodies between the two-time points. Each color represents one pregnant individual. **E** Anti-N and anti-RBD IgA antibody levels in paired maternal and cord blood at delivery. **F** Anti-N and anti-RBD IgG antibody levels in paired maternal and cord blood at delivery. The dotted line represents the positive cutoff value. **G** Levels of IgA in plasma (left *y*-axis) and breastmilk (right *y*-axis) in five lactating individuals after the third dose of vaccination. Plasma and breast milk samples were collected at the same time. **H** Correlation of IgA in plasma and breastmilk.

We next investigated the kinetics of IgA production and decay in our cohort. Breakthrough infection generally induced higher anti-RBD IgA levels than 3-dose vaccination. No significant difference in overall level was observed for both anti-RBD and anti-N IgA antibodies up to 100 days after exposure (Fig. 6A, B). To examine the rate of IgA decay in maternal plasma, we compared levels after breakthrough infection with levels at the time of delivery. Anti-N IgA antibodies at delivery dropped significantly from 12-31 days post-infection (4.6-fold, *P* = 0.03), while the anti-RBD IgA antibody levels remained stable over the same time period (1.5-fold, *P* = 0.65) (Fig. 6C, D). In these participants, anti-N IgA decayed faster than anti-RBD IgA (relative decay rate 0.79 vs 0.23, *P* = 0.02). At the time of delivery, only 2 of 6 had detectable levels of anti-N IgA in maternal plasma; in comparison, all participants had detectable anti-RBD IgA. As expected, neither anti-N nor anti-RBD IgA antibodies were detected in cord blood (Fig. 6E); in contrast, IgG antibodies were efficiently transferred (Fig. 6F).

Paired breast milk and plasma samples were collected from 5 lactating participants after the third dose of vaccination (Fig. 6G). In 4 of 5 participants, IgA levels were comparable between matched milk and plasma samples. However, there was no statistically significant correlation between milk and plasma IgA levels (Fig. 6H).

## Discussion

In pregnancy, hybrid immunity induced the highest nAbs levels compared to 2- or 3-dose vaccination alone or infection alone. This is similar to other reports in non-pregnant populations^39–43^. Our results provide a biological basis for the results of a recent clinical study that showed maternal hybrid immunity provided young infants the best protection against COVID-19 hospitalizations when compared to vaccination or infection alone^7^. Vaccine-induced antibodies were more efficiently transferred to the fetus than infection-induced antibodies; infants of vaccinated mothers had higher cord antibody levels than infants of infected mothers. Although vaccination and infection with the WT variant induced very low levels of anti-Omicron nAbs, the transfer ratios did not differ significantly. Interestingly, there appears to be individual-level variability in the transfer efficiency of variant-specific nAbs, which warrants further study.

Antibodies against N and S are the most sensitive and commonly measured targets of immunity^44,45^. They can be used to distinguish between natural immunity (anti-N and anti-S) from vaccine-induced immunity (anti-S only)^17,46^. In infection after prior vaccination, circulating antibodies and SARS-CoV-2-specific CD8 + T cells may increase the rate of viral clearance early in infection^47^, which may decrease primary exposure to N antigen and result in lower anti-N IgG. In our study, although there was a trend toward decreased anti-N IgG in individuals after breakthrough infection compared to naïve infection (Fig. 4A), however, this was not statistically significant. Additional studies are needed to assess the accuracy of anti-N and anti-S IgG as correlates of protection against SARS-CoV-2 in the era of widespread hybrid immunity.

We show that nAb against the WT and Delta variants tended to increase with higher anti-RBD IgG levels, which is consistent with nAb targeting the RBD on Spike protein to block binding to host cell ACE2 receptors. However, the only statistically significant correlation was between anti-WT nAb and anti-RBD-IgG in breakthrough infection. (Fig. 4). This is consistent with another study that demonstrated that IgG assays may not accurately predict neutralizing activity in a non-pregnant cohort^44^. This discrepancy may be due to the contributions of non-IgG class antibodies on plasma neutralizing activity, as optimal neutralizing activity is achieved when all three immunoglobulin classes (IgG, IgM, and IgA) are present^48^. Furthermore, in vivo, non-nAbs have been shown to play diverse and important roles in immune responses, including opsonization, mediating antibody-dependent cellular cytotoxicity (ADCC), complement activation, promoting clearance in the reticuloendothelial system through immune complex formation, and regulating immune responses via the interaction with the Fc receptor^49–53^. These activities do not directly neutralize pathogens through antigen-antibody reactions, which may complicate IgG assays and the interpretation of neutralizing activity. Our findings did not reveal any correlation between IgA levels and either IgG or nAb against three variants (Supplementary Fig. 4), although generalizability may be limited due to the small sample size.

The anti-RBD IgA response after breakthrough infection was greater than after 3-dose vaccination, which is consistent with data from a non-pregnant cohort^54^. A prior longitudinal study showed that IgA has similar kinetics of induction and time to peak levels to IgG but with more rapid decay to low levels by 4 weeks post-infection than Spike-specific IgG^55^. In contrast, we found that anti-RBD IgA was resistant to decay, with stable levels up to 200 days after breakthrough infection (Fig. 6D). The decay of anti-N IgA was faster, with 50% of cases undetectable by 178 days (Fig. 6C). One potential mechanism for the increased durability of anti-RBD IgA is the generation of RBD-specific memory B cells after mRNA vaccination, which have been shown to continue to increase in frequency from 3 months to 6 months post-vaccination^56^. The sustained anti-RBD IgA antibodies throughout pregnancy may benefit breastfed infants by providing them with mucosal protection against SARS-CoV-2 through breast milk^22^. More study is needed on the kinetics of B-cell responses, IgA induction, and the durability of responses in pregnancy, particularly to aid in the development of mucosal vaccine strategies for the mother-infant dyad.

Functional SARS-CoV-2 antibodies elicited by vaccination or infection can efficiently transfer to the fetus and are equally competent in both maternal and cord blood^57,58^. In our analysis comparing vaccinated vs infected maternal-cord dyads, matched by gestational age of exposure, vaccination was found to be associated with higher nAb levels in cord blood resulting from a higher transfer ratio. One potential explanation is alterations in glycosylation profiles in vaccination, which has been shown to influence maternal-fetal antibody transfer^59–61^. However, nAb against Omicron was low to undetectable in maternal blood and absent in the majority of cord blood samples, consistent with other reports^62–64^. With the high mutation rate of SARS-CoV-2 resulting in rapid changes in the circulating variants, further studies are needed to assess the ability of the existing vaccine formulations, including the currently administered Omicron BA.4/BA.5 bivalent or XBB.1.5 monovalent vaccine, to provide cross-variant protection to the mother-infant dyad.

Our study has several limitations. First, this is a small cohort in a highly vulnerable population. However, detailed studies of pregnant individuals are needed to dissect the intricate humoral immune responses to SAR-CoV-2 within the altered immune state of pregnancy. Second, this study is focused on the binding properties of antibodies generated after vaccination or infection in pregnancy. Vaccines designed to induce high levels of nAbs are effective in preventing COVID-19, highlighting the crucial role of neutralizing antibody acquisition in immune defense. However, as noted above, in addition to the direct neutralizing activity of antibodies, many other protective immune responses are triggered by antibody interactions with Fc receptors^49,53,65^. Additional studies are needed to increase our understanding of whether Fc effector functions are altered in pregnancy or lactation^66,67^.

Strengths of our study include the diverse cohort of pregnant individuals with varying exposures to SARS-CoV-2. This provides a comprehensive investigation of antibody responses after vaccine-induced, infection-induced, and hybrid immunity. We also examined the relationships between neutralizing and binding antibodies, which are often assumed to be interchangeable in reports of SARS-CoV-2 humoral immunity. Finally, we provide the first detailed study of SARS-CoV-2 specific IgA responses in pregnancy, which are important for maternal-infant immune transfer through breast milk.

In conclusion, our results demonstrated that breakthrough infection results in the highest breadth and magnitude of neutralizing response in pregnant individuals. Vaccine-induced antibodies (both binding and neutralizing) were more efficiently transferred to the infants compared to those induced by infection. We also describe, for the first time, the durability of anti-RBD IgA antibodies induced by hybrid immunity. This sustained IgA response may be a mechanism to provide additional protection to the infant through breastmilk secretion. Future studies identifying immune correlates of optimal protection against SARS-CoV-2 are needed to guide the design of future vaccines that maximize protection for the mother-infant dyad.

## Methods

### Participants and blood sampling

All participants in this study enrolled in two COVID-19-related cohorts evaluating SARS-CoV-2 infection and mRNA vaccination in pregnant individuals at the University of California, San Francisco. This study was approved by the institutional review board of the UCSF (IRB# 19-29713; #20-32077), Santa Clara Valley Medical Center (IRB# 20-021), Oregon Health and Sciences University (IRB# STUDY00021569), and Marshall University (IRB# 1662248-1). Informed consent was obtained from all participants. In the vaccination cohort (enrolled between December 2020–February 2022), participants received at least one dose of the COVID-19 mRNA vaccine (BNT162b2 (Pfizer-BioNTech) or mRNA-1273 (Moderna)) during pregnancy and were followed prospectively. Longitudinal blood sampling was performed at the time of additional doses, after infection, and/or at the time of delivery. The infection-only (naïve infection) group was enrolled during the initial COVID-19 pandemic (March 2020–December 2020) before vaccines were available in early 2021. Breakthrough infections refer to infections that occur in individuals after two or three doses of vaccine. Nonpregnant and non-lactating women infected prior to any vaccine dose (*n* = 6) or infected after three doses (*n* = 3) during the Omicron wave were included as controls. All participants received the original monovalent vaccines.

Pregnant or lactating individuals were organized into 8 groups (Fig. 1): (1) naïve infection without prior vaccination; (2) original 2-dose regimen; (3) third dose in pregnancy, with first two doses prior to conception; (4) third dose in lactation, with first two doses in pregnancy; (5) breakthrough in pregnancy after two doses; (6) breakthrough infection in pregnancy after three doses; (7) 3-dose vaccination in non-pregnant non-lactating individuals; and (8) naïve infection in non-pregnant non-lactating individuals. Blood was collected at a median of 34.5 days (ranging from 9 days to 79 days) after the last exposure (vaccination or infection). In an additional set of 30 pregnant participants with 2-dose vaccination and 30 gestational age-matched pregnancies with naïve infection (Fig. 2A), paired maternal and cord blood was collected at the time of delivery as previously described^58^. Plasma was isolated and cryopreserved at −80 °C until use.

### Breast milk collection and IgA measurement

Breast milk samples were collected concurrently with blood samples from lactating individuals after the third vaccine dose. IgA levels in the breast milk were assessed using an anti-Spike ELISA assay (Euroimmune, Germany) as previously described^22^. Briefly, milk fat was removed via cold centrifugation and diluted at a 1:4 ratio with the provided diluent buffer. The samples were added to the ELISA plate after blocking with 5% BSA in TBS with 0.5% Tween 20 for 30 min to enhance specificity. Optical density (OD) values of the samples were determined by dividing by the calibrator OD value supplied with the kit; samples with a sample ratio greater than 1 were considered positive.

### Pseudotyped virion nAb assay

For the preparation of virions, 293T cells were transfected with the Spike plasmid, followed by inoculation with a previously generated working stock of rVSVΔG-rLuc*G (G protein-deficient vesicular stomatitis virus) containing an integrated Renilla luciferase reporter gene) to generate the pseudotyped rVSVΔG-rLuc*SARS-CoV-2. Pseudotyped virions were generated using Spike plasmids harboring mutations found in the WT SARS-CoV-2 Spike (Wuhan-Hu-1; GenBank accession number MN908947.3), the Delta variant (T19R, G142D, E156 deletion, F157 deletion, R158G, L452R, T478K, D614G, P681R, and D950N), and the Omicron BA.1 variant (S371L, S373P, S375F, K417N, N440K, G446S, S477N, T478K, E484A, Q493K, G496S, Q498R, N501Y, and Y505H). The three types of virions were titrated based on the TCID50 method and the infectivity titers were equalized. To determine the neutralization activity of plasma, pseudotyped virion nAb experiments were performed with Calu-6 epithelial cells (ATCC HTB56) stably expressing human angiotensin-converting enzyme 2 (hACE2; OriGene, RC08442). Twenty-four hours before administration of virion, 2.5 × 10^4^ Calu-6-hACE2 cells were plated per well of a 96-well plate in 200 μL of complete DMEM. The SARS-CoV-2 Spike-pseudotyped virions harvested from the supernatant of the 293T cells were assayed for titration and then aliquots were mixed for 30 min with heat-inactivated plasma samples. Plasma samples were diluted in calcium-free DMEM starting at a 1:15 dilution, and then at 3-fold serial dilutions for six final concentrations, in triplicate. The mixtures were then used to infect Calu-6-hACE2 cells and incubated at 37 °C and 5% CO_2_ for 24 h. At 24 h after infection, the cells were washed once with 1× PBS, then 20 μL of lysis buffer was added per well, followed by 100 μL of Renilla luciferase substrate/buffer (Promega, E2810) according to the manufacturer’s instructions. The plates were read on a luminometer. SARS-CoV-2 NT50 titers of the plasma samples were defined as the sample dilution at which a 50% reduction was observed relative to the average of the virus control wells. All NT50 titers were calculated as an average of three independent experiments. The composite NT50 value was calculated by averaging the NT50 values of the WT strain and the two variants.

### Multiplex bead-based assay of SARS-Cov-2 binding antibodies

IgG and IgA antibodies against the RBD and N of the SARS-CoV-2 virus were analyzed with a multiplex-based human serology kit (Bio-Rad, CA, USA) according to the manufacturer’s instructions. Magnetic beads conjugated with RBD and N protein were available for purchase from Bio-Rad. Briefly, plasma samples were diluted at 1:1000 for IgG detection and 1:100 for IgA detection. Diluted samples were incubated with coupled beads for 30 minutes at room temperature (RT). Secondary antibodies for IgG and IgA were added into respective wells and incubated for 30 min at RT, followed by 10 min incubation with the streptavidin-phycoerythrin. After proper wash and resuspension of the beads, the reactions were read on a BioPlex-200 (Bio-Rad), and the results were expressed as median fluorescence intensity (MFI) per 100 beads. The maximum MFI of our assay was 25,000, determined by measuring undiluted plasma as a positive control (data not shown). To determine the positive cut-off values of the multiplex platform, we performed a comparative analysis of anti-SARS-CoV-2 IgG on previously vaccinated and infected samples with known quantitative antibody results provided by previous analysis using the clinically validated Pylon 3D automated immunoassay system^58,68,69^.

### Bulk SARS-CoV-2–specific IgG antibodies measurement

Bulk anti-RBD and anti-N binding IgG in paired maternal and cord plasma collected at delivery were measured using the Pylon 3D automated immunoassay system (ET Healthcare, Palo Alto, CA) as previously described^68,69^. The background-corrected signal of SARS-CoV-2 specific IgM and IgG antibodies was reported as relative fluorescent units (RFU); measurements higher than 50 RFU were considered positive.

### Statistical analysis

Statistical analysis was performed on GraphPad Prism 9 (GraphPad, CA, USA). Results are presented as median ± interquartile range (IQR). Significance between two groups was assessed using the Mann–Whitney test, or Wilcoxon signed-rank test for matched maternal-cord pairs. The Kruskal–Wallis test with Dunn’s multiple comparisons test was used for comparisons among more than two groups. The relative decay rate of antibodies was calculated by dividing the percentage decrease ([starting value − final value]/starting value × 100) by duration (days). Correlations between nAbs with IgG or IgA, and IgG with IgA were reported using Spearman’s rank correlation test. Differences were considered statistically significant when *P* < 0.05.

### Supplementary information

Supplementary Information

## Data Availability

All data generated or analyzed during this study are included in this published article and its supplementary information files.
