# Supplementary material for: Neutralizing and binding antibody responses to SARS-CoV-2 with hybrid immunity in pregnancy
Source: NPJ Vaccines. 2024 Aug 27;9:156. doi: 10.1038/s41541-024-00948-3 (PMC11349990; doi:10.1038/s41541-024-00948-3)
Supplement: Supplementary file 1 — Supplementary Information [file 41541_2024_948_MOESM1_ESM.pdf]

Neutralizing and binding antibody responses to SARS-CoV-2 with hybrid immunity in pregnancy

Supplementary Figures

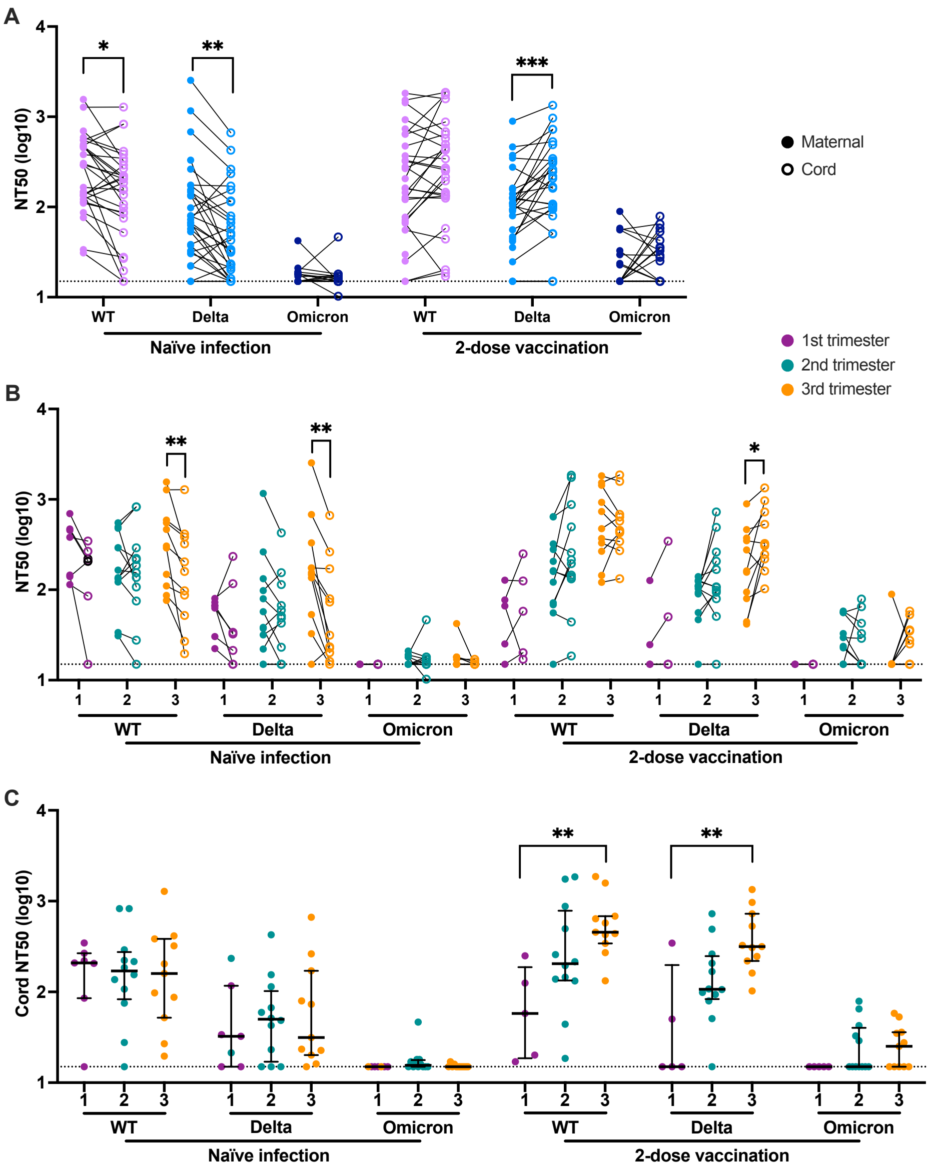

Supplementary Fig. 1: Neutralizing activities in maternal and cord blood in pregnant individuals with naïve infection and original 2-dose vaccination

A. NAb against the WT, Delta, and Omicron variants in matched maternal and cord blood at delivery. Lines connect the maternal-cord blood dyads.
B. NAb against each variant was further divided by trimesters at the time of first exposure.
C. Cord nAb levels against three variants

The black dotted line represents the positive cutoff value of 15. The black error bars depict the median ± interquartile range (IQR). **P* < 0.05, ***P* < 0.01, ****P* < 0.001 by Wilcoxon matched-pairs signed-rank test or Mann-Whitney test.

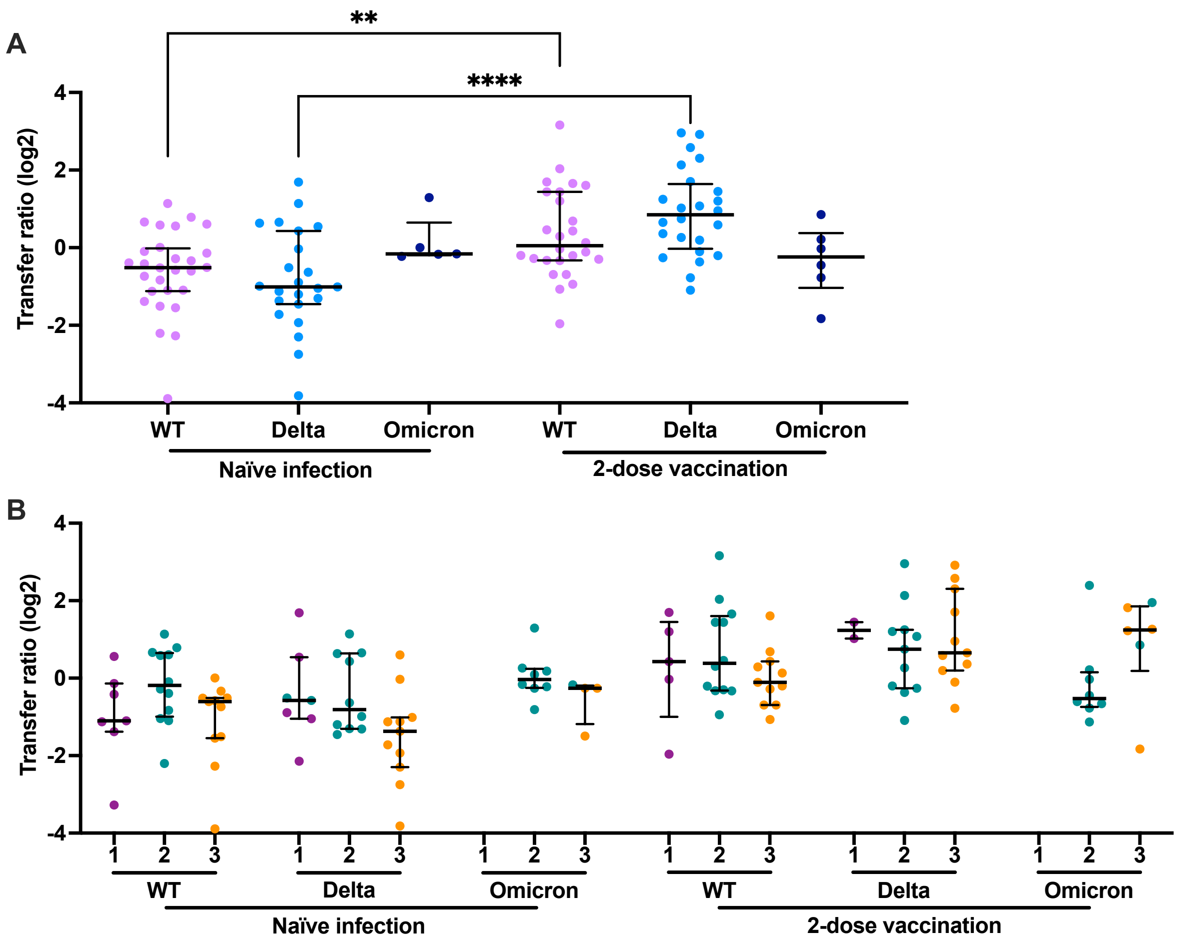

Supplementary Fig. 2: Maternal-fetal transfer ratio

A. The maternal-cord blood transfer ratio (log2) of nAb against the WT, Delta, and Omicron variants was calculated by cord NT50 divided by maternal NT50.
B. Maternal-cord blood transfer ratio (log2) of each variant is shown by trimesters.
The black error bars depict the median ± interquartile range (IQR). $^{**}P < 0.01$, $^{****}P < 0.0001$ by Mann-Whitney test.

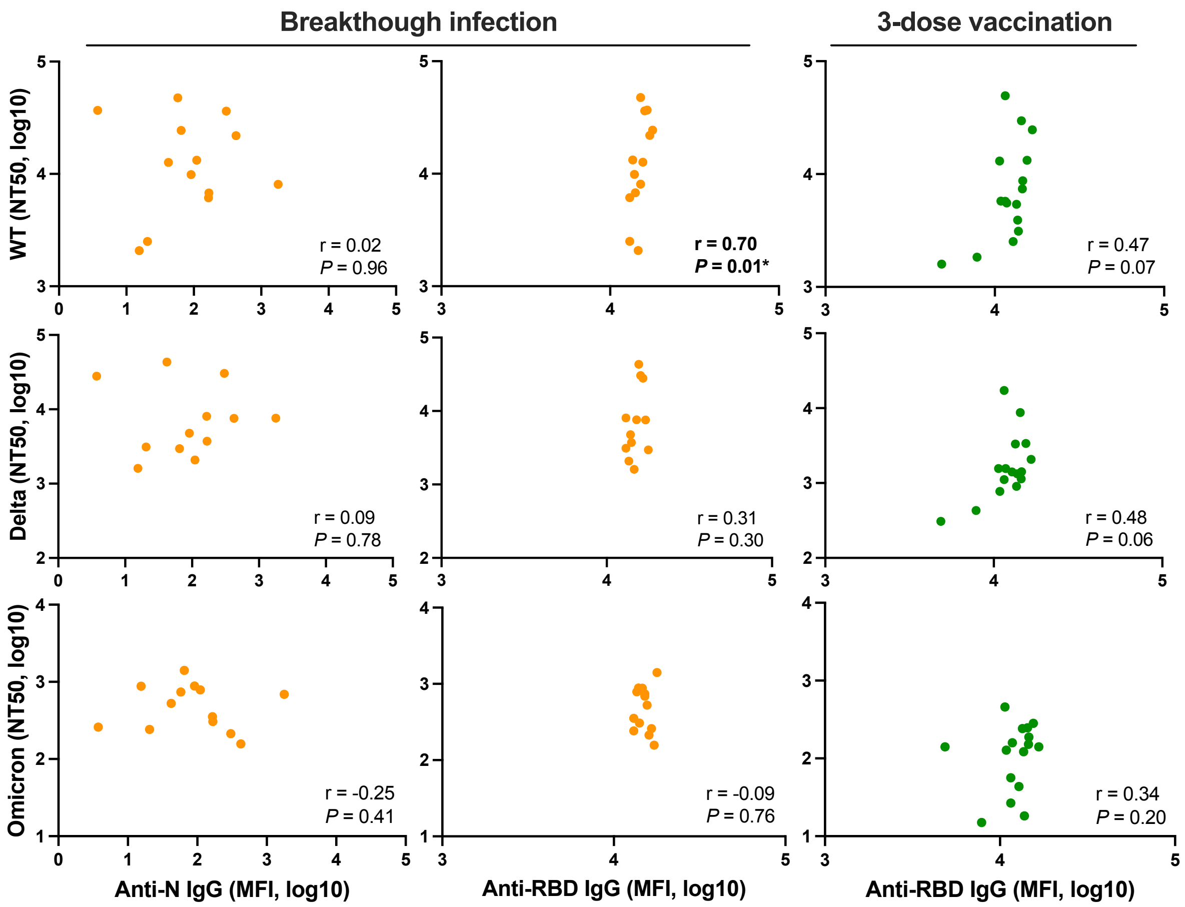

Supplementary Fig.3 Correlation analysis of anti-N and anti-RBD IgG with NT50 against each SARS-CoV-2 variant after breakthrough infection or 3-dose vaccination, respectively. * $P < 0.05$ by Spearman's rank correlation.

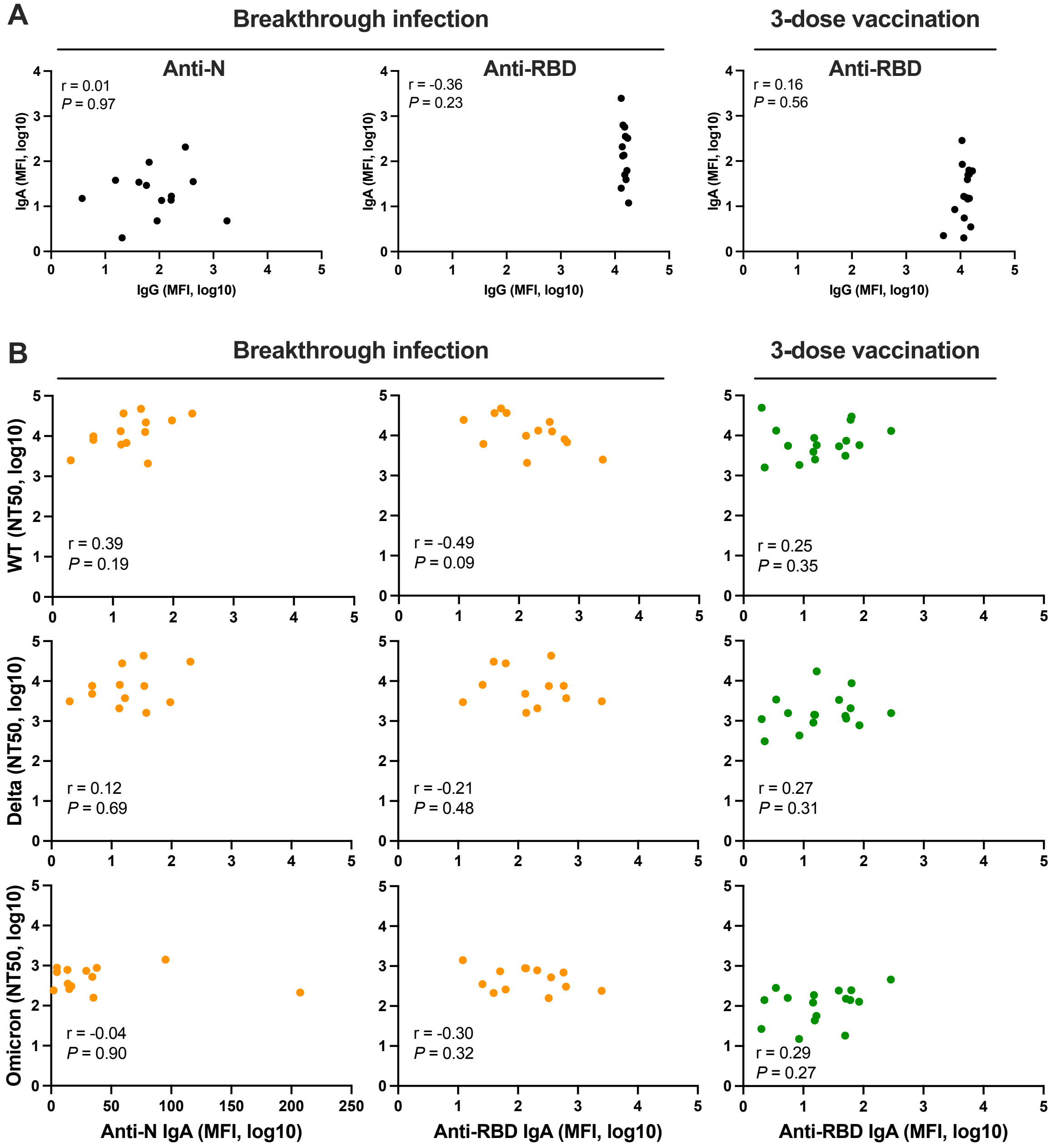

Supplementary Fig.4: Correlation analysis of IgA with IgG and neutralizing antibodies.

A. Correlation between IgA and IgG against N and RBD after breakthrough infection and 3-dose vaccination.
B. Correlation analysis of anti-N or anti-RBD IgA with NT50 against each SARS-CoV-2 variant after breakthrough infection and 3-dose vaccination, respectively.

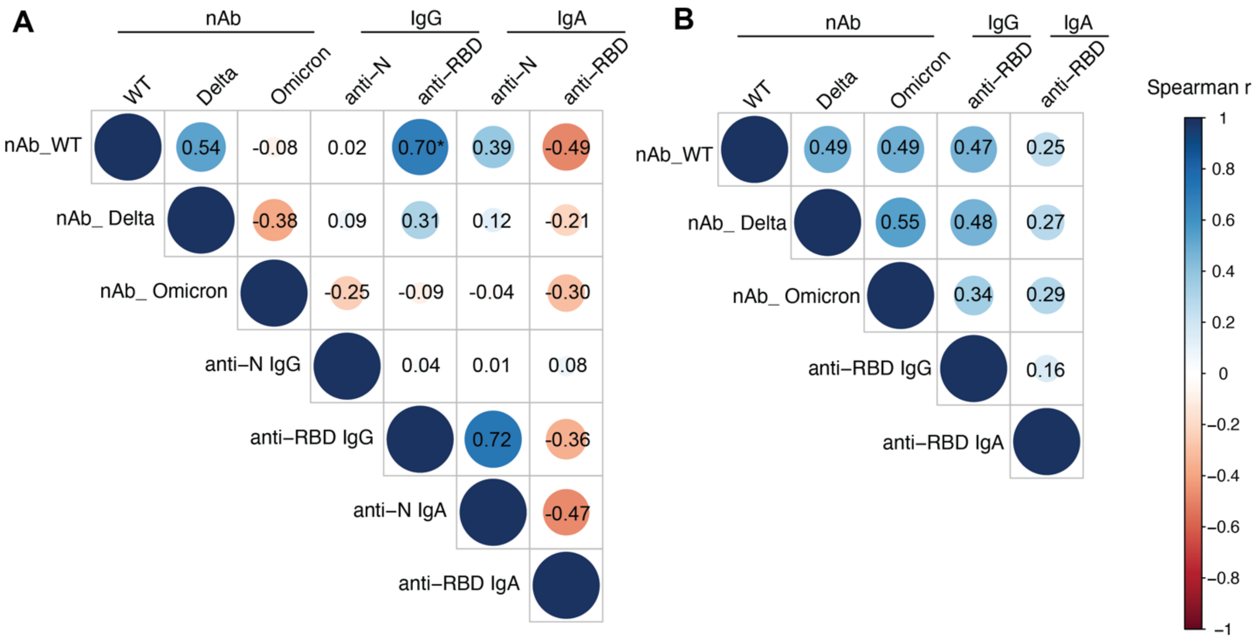

Supplementary Fig 5: Correlation matrix of neutralizing antibodies, IgG, and IgA.

A. Breakthrough infection.
B. 3-dose vaccination.
* *P* < 0.05 by Spearman's rank correlation.
